# Cystic Fibrosis Isolates of *Pseudomonas aeruginosa* Retain Iron-Regulated Antimicrobial Activity against *Staphylococcus aureus* through the Action of Multiple Alkylquinolones

**DOI:** 10.3389/fmicb.2016.01171

**Published:** 2016-07-27

**Authors:** Angela T. Nguyen, Jace W. Jones, Miguel Cámara, Paul Williams, Maureen A. Kane, Amanda G. Oglesby-Sherrouse

**Affiliations:** ^1^Department of Pharmaceutical Sciences, School of Pharmacy, University of MarylandBaltimore, MD, USA; ^2^Centre for Biomolecular Sciences, School of Life Sciences, University of NottinghamNottingham, UK; ^3^Department of Microbiology and Immunology, School of Medicine, University of MarylandBaltimore, MD, USA

**Keywords:** *Pseudomonas aeruginosa*, iron regulation, AQs, *Staphylococcus aureus*, quorum sensing

## Abstract

Cystic fibrosis (CF) is a hereditary disease that predisposes individuals to pulmonary dysfunction and chronic infections. Early infection of the CF lung with *Staphylococcus aureus* is common, while *Pseudomonas aeruginosa* becomes dominant as disease progresses. Emergence of *P. aeruginosa* likely depends on the action of multiple 2-alkyl-4-(1*H*)-quinolones (AQ) secreted by this organism. We recently showed that antimicrobial activity against *S. aureus* is enhanced by iron depletion and is dependent upon multiple AQ metabolites. Two of these AQs, the Pseudomonas quinolone signal [PQS; 2-heptyl-3-hydroxy-4(1*H*)-quinolone] and 2-heptyl-4-hydroxyquinoline (HHQ), are quorum sensing molecules that activate the expression of multiple microbicidal factors. Here we show for the first time that HHQ also exhibits innate antimicrobial activity against *S. aureus*. We further show that iron depletion potentiates the antistaphylococcal activity of HHQ, as well as 2-heptyl-4-hydroxyquinoline-*N*-oxide (HQNO), another AQ that functions as a cytochrome B inhibitor. Notably, we found that deletion of the genes for the terminal biosynthetic steps for either PQS or HQNO results in overproduction of the HHQ intermediate, likely maintaining the ability of these mutants to mediate antimicrobial activity. Compensatory increases in HHQ were also observed in PQS-deficient CF isolates, which also retained the ability to mediate iron-regulated antimicrobial activity against *S. aureus*. These studies demonstrate that iron-regulated antimicrobial activity of *P. aeruginosa* against *S. aureus* is due to the cumulative effects of multiple AQ metabolites, both the production and activity of which are modulated by environmental iron levels.

## Introduction

CF is a hereditary disease that predisposes patients to pulmonary dysfunction and chronic infection by multiple microorganisms, including *Staphylococcus aureus* and *Pseudomonas aeruginosa*. While *S. aureus* is one of the dominant pathogens during early CF lung infection, *P. aeruginosa* eventually becomes the predominant pathogenic resident in the CF lung and persists for decades as a chronic infection (Hoiby et al., 2010; Cystic Fibrosis Foundation, 2014). The factors that contribute to this shift are not well understood, but isolation of *P. aeruginosa* from the lungs of CF patients is correlated with increased exacerbation and a decline in lung function (Rabin et al., 2004; Bhatt, 2013). Thus, understanding the factors that contribute to long term survival of *P. aeruginosa* in the CF lung is essential to understanding disease progression in these patients.

**Figure 1:**
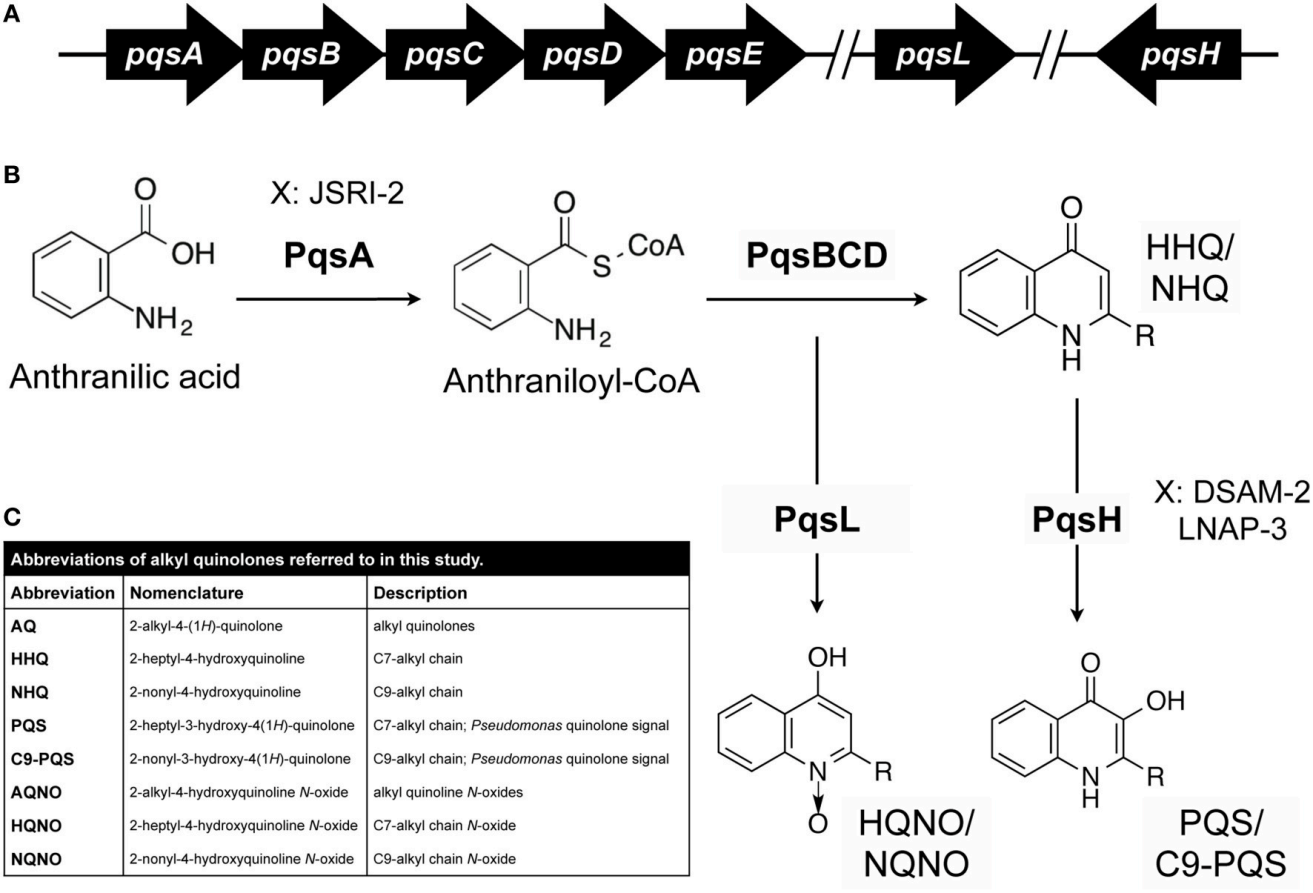
**AQ biosynthesis in *P. aeruginosa*. (A,B)** Genetics of AQ biosynthesis. Anthranilic acid is converted to anthraniloyl-CoA by PqsA. The genes encoding PqsBCD, which contributes to the production of HHQ and congeners, are located in an operon with PqsA. The PqsL and PqsH enzymes, which mediate synthesis of HQNO and PQS respectively, and related congeners are encoded at distal sites of the chromosome. Each of these AQ core structures varies with respect to the length and saturation of their alkyl chain (–R). Specific CF isolates of *P. aeruginosa* (JSRI-2, DSAM-2, and LNAP-3) are hypothesized to defective at distinct steps in the AQ metabolic pathway as indicated. **(C)** AQ abbreviations used in this study.

2-alkyl-4-(1*H*)-quinolones (AQs) produced by *P. aeruginosa* are hypothesized to contribute to the eventual dominance of this pathogen in the CF lung (Mashburn et al., 2005). AQ production requires the co-enzyme ligase PqsA, which converts a cellular metabolite, anthranilate, to its active form, anthraniloyl CoA (Figure 1; Coleman et al., 2008). This in conjunction with fatty acids and the PqsBCD enzyme complex results in the production of over 55 distinct AQs (Coleman et al., 2008; Dulcey et al., 2013; Drees et al., 2016). AQs exhibit a variety of functions including cell signaling, redox activity, and antimicrobial activity (Deziel et al., 2004; Diggle et al., 2007). Perhaps the best studied AQs are 2-heptyl-4-hydroxyquinoline (HHQ; Wratten et al., 1977) and the Pseudomonas quinolone signal [PQS; 2-heptyl-3-hydroxy-4(1*H*)-quinolone; Calfee et al., 2001], which function as quorum signaling molecules and induce the expression of virulence-associated genes. Included in the PQS/HHQ regulon are genes required for the production of exoenzymes, lectins, siderophores (pyochelin and pyoverdine), and phenazines (Dietrich et al., 2006; Diggle et al., 2007; Rampioni et al., 2010). Phenazines are redox active metabolites produced by *P. aeruginosa* that generate reactive oxygen species resulting in the lysis of both human and microbial cells (Price-Whelan et al., 2006). Exoenzymes such as elastases contribute to the lysis of *S. aureus* by cleaving the peptidoglycan pentaglycine interpeptides of the cell wall (Kessler et al., 1993, 1997). The PqsABCD biosynthetic pathway in conjunction with the mono-oxygenase PqsL (Heeb et al., 2011) can also generate AQ N-oxides (AQNOs) such as 2-heptyl-4-hydroxyquinoline-*N*-oxide (HQNO), which suppresses growth of gram-positive bacteria such as *S. aureus* (Figure 1; Van Ark and Berden, 1977; Machan et al., 1992; Smirnova et al., 1995; Rothery and Weiner, 1996; Hoffman et al., 2006).

Another major factor in *P. aeruginosa's* ability to survive in the complex microbial environment of the CF lung is the acquisition of essential nutrients, including iron. *P. aeruginosa* requires iron for growth and virulence (Meyer et al., 1996; Takase et al., 2000; Xiong et al., 2000; Nadal Jimenez et al., 2010), yet this element is sequestered during infection by multiple host proteins (Otto et al., 1992; Nairz et al., 2010). To obtain iron from the host, *P. aeruginosa* expresses multiple high affinity iron uptake systems, which have been studied extensively over the past three decades (Cornelis, 2010; Cornelis and Dingemans, 2013; Konings et al., 2013). Previously, it was reported that *P. aeruginosa* could also lyse and acquire iron from *S. aureus* through the secretion of AQs (Mashburn et al., 2005), the production of which is stimulated by peptidoglycan released from *S. aureus* upon cell lysis (Korgaonkar et al., 2013). A recent report also showed that the acquisition of iron by siderophores is required for efficient killing of *S. aureus* by HQNO in both planktonic and biofilm culture environments (Filkins et al., 2015). Coinciding with this report, we showed that production of a PQS congener with a longer alkyl chain is enhanced by iron depletion, correlating with increased antimicrobial activity against *S. aureus* in iron-depleted environments (Nguyen et al., 2015). Combined, these studies demonstrate a complex relationship between iron and AQ-mediated interactions of *P. aeruginosa* and *S. aureus*.

In the current work, we sought to determine how individual AQs produced by *P. aeruginosa* contribute to iron-regulated antimicrobial activity against *S. aureus*. We show that, in addition to its roles as a quorum sensing molecule, HHQ exhibits innate antimicrobial activity against *S. aureus*. We further show that iron depletion potentiates the innate antimicrobial activity of HHQ and HQNO against *S. aureus*. Despite significant changes in AQ metabolites incurred by clonal, longitudinal CF isolates, we demonstrate that iron-regulated antimicrobial activity against *S. aureus* is largely retained by these isolates. We postulate that conservation of this phenomenon is due to the cumulative effects of multiple AQ metabolites, both the production and activity of which are modulated by environmental iron levels.

## Materials and methods

### Strains and growth media

*P. aeruginosa* and *S. aureus* strains used in this study are shown in Table 1. CF isolates of *P. aeruginosa* were originally obtained from Dr. David Speert and previously described by our lab (Nguyen et al., 2014). Brain heart infusion (BHI) was used for routine culture of *S. aureus* and *P. aeruginosa*. Dialyzed trypticase soy broth (DTSB) for iron studies was prepared as previously described for iron-depleted medium (Oglesby-Sherrouse et al., 2014). Cultures were supplemented with 100 μM FeCl_3_ as indicated.

**Table 1:** **Strains used in this study**.

**Strain**	**Description**	**Sources**
PAO1	Wild type *P. aeruginosa* strain used for mutational analysis in this and previous studies.	Holloway, 1955
Δ*pqsA*	Deletion of *pqsA* gene generated in PAO1.	Nguyen et al., 2014
Δ*pqsL*	Deletion of *pqsL* gene generated in PAO1.	D'Argenio et al., 2002
PA14	Burn wound isolate from 1995 at the Massachusetts General Hospital, Boston.	Rahme et al., 1995
Δ*pqsH*	Deletion of *pqsH* gene generated in PA14.	Nguyen et al., 2015
JSRI-1	CF *P. aeruginosa* lung isolate from 8 year-old patient.	Nguyen et al., 2014
JSRI-2	CF *P. aeruginosa* lung isolate from 17 year-old patient.	Nguyen et al., 2014
DSAM-1	CF *P. aeruginosa* lung isolate from 11 year-old patient.	Nguyen et al., 2014
DSAM-2	CF *P. aeruginosa* lung isolate from 18 year-old patient.	Nguyen et al., 2014
DSAM-3	CF *P. aeruginosa* lung isolate from 22 year-old patient.	Nguyen et al., 2014
LNAP-1	CF *P. aeruginosa* lung isolate from 2 year-old patient.	Nguyen et al., 2014
LNAP-2	CF *P. aeruginosa* lung isolate from 13 year-old patient.	Nguyen et al., 2014
LNAP-3	CF *P. aeruginosa* lung isolate from 19 year-old patient.	Nguyen et al., 2014
MRSA-M2	Methicillin-resistant isolate of *S. aureus* isolated from an osteomyelitis patient in Galveston, Texas.	Harro et al., 2013

### Antimicrobial assays

To prepare extracts for antimicrobial assays, *P. aeruginosa* strains were grown in DTSB medium supplemented with or without 100 μM FeCl_3_ for 18 h at 37°C. OD_600_ of cultures was measured and cultures were centrifuged for 5 min at 14,000 RPM (~16,000 × g) in a tabletop centrifuge. Supernatant volume collected for extraction was normalized to the lowest culture OD in each biological replicate. Supernatants were extracted as previously described (Collier et al., 2002) and 1 ml was evaporated to dryness. Dried extracts were resuspended in 20 μL of 100% ethyl alcohol. For antimicrobial assays, the methicillin-resistant *S. aureus* strain M2 was grown for 18 h in BHI medium at 37°C. Growth was measured by OD_600_, then diluted to an OD 0.05 in DTSB medium supplemented with or without 100 μM FeCl_3_. 1 μL of *P. aeruginosa* extracts was added to 200 μL of diluted *S. aureus* culture. 1 μL 100% ethyl alcohol was used as a solvent control. Cultures were incubated for 18 h with shaking at 37°C in a 96-well plate, and *S. aureus* cell density (OD_630_) was measured spectroscopically in a BioTek® Synergy™ HT plate reader.

### Mass spectrometric quantification of AQs

Strains were grown for 18 h in DTSB supplemented with or without 100 μM FeCl_3_ as indicated. Cells were spun down and supernatants harvested for AQ extraction as previously described by Collier et al. (2002). Nalidixic acid and deuterated *N*-dodecanoyl-L-homoserine lactone (C_12_-HSL) were used as internal standards. LC-MS/MS analysis was performed as previously described (Ortori et al., 2011; Nguyen et al., 2015).

### Transwell co-culture assay

To quantify antimicrobial activity against *S. aureus*, a liquid co-culture system using transwell cell culture inserts (Corning Costar®, NY, USA) was performed as previously described (Nguyen et al., 2015). Briefly, *P. aeruginosa* and *S. aureus* strains were grown overnight in DTSB for 18 h at 37°C. *S. aureus* cultures were diluted to an OD_600_ of 0.05 in DTSB supplemented with or without 100 μM FeCl_3_, and 600 μL of the resulting cell suspension was inoculated into the bottom of the transwell plate. A transwell insert with a 0.4 μm membrane was then placed onto the plate, and 100 μL of *P. aeruginosa* cultures, diluted to an OD_600_ of 0.05, were inoculated on top of the membrane. The transwell plates were incubated at 37°C for 18 h under static growth conditions, and *S. aureus* cell density (OD_630_) was measured spectroscopically in a BioTek® Synergy™ HT plate reader.

### Real time PCR analysis

Real time PCR (qPCR) analysis of *P. aeruginosa* and *S. aureus* gene expression in broth cultures was carried out as previously described (Oglesby et al., 2008; Nguyen et al., 2014; Reinhart et al., 2015), using the Applied Biosystems StepOne Plus Real Time PCR System (Life Technologies). Primers and probes used in this study are listed in Table S1. For *S. aureus* cell lysis: cell pellets were resuspended in 100 μL of 2.5 μg/μL lysozyme and 0.25 μg/μL lysostaphin in TE and incubated for 45 min at 37°C. Relative amounts of cDNA were determined by the ΔΔC_T_ method, and expression was normalized to *oprF* cDNA for *P. aeruginosa* or *rpoB* cDNA for *S. aureus* detected in each sample.

### Thin layer chromatography (TLC)

Bacteria were grown in DTSB for 18 h at 37°C, with or without 100 μM FeCl_3_ supplementation as indicated. Each culture was harvested and extracted with acidified ethyl acetate as described by Collier et al. (2002). One half of the resulting organic extract was transferred to a clean tube and evaporated to dryness. Samples were resuspended in 1:1 acidified ethyl acetate:acetonitrile and analyzed by thin-layer chromatography (TLC) with a synthetic PQS standard (Pesci et al., 1999).

## Results

### Antimicrobial activity of alkylquinoline-*N*-oxides (AQNOs) is dependent on *S. aureus* growth environment

**Figure 2:**
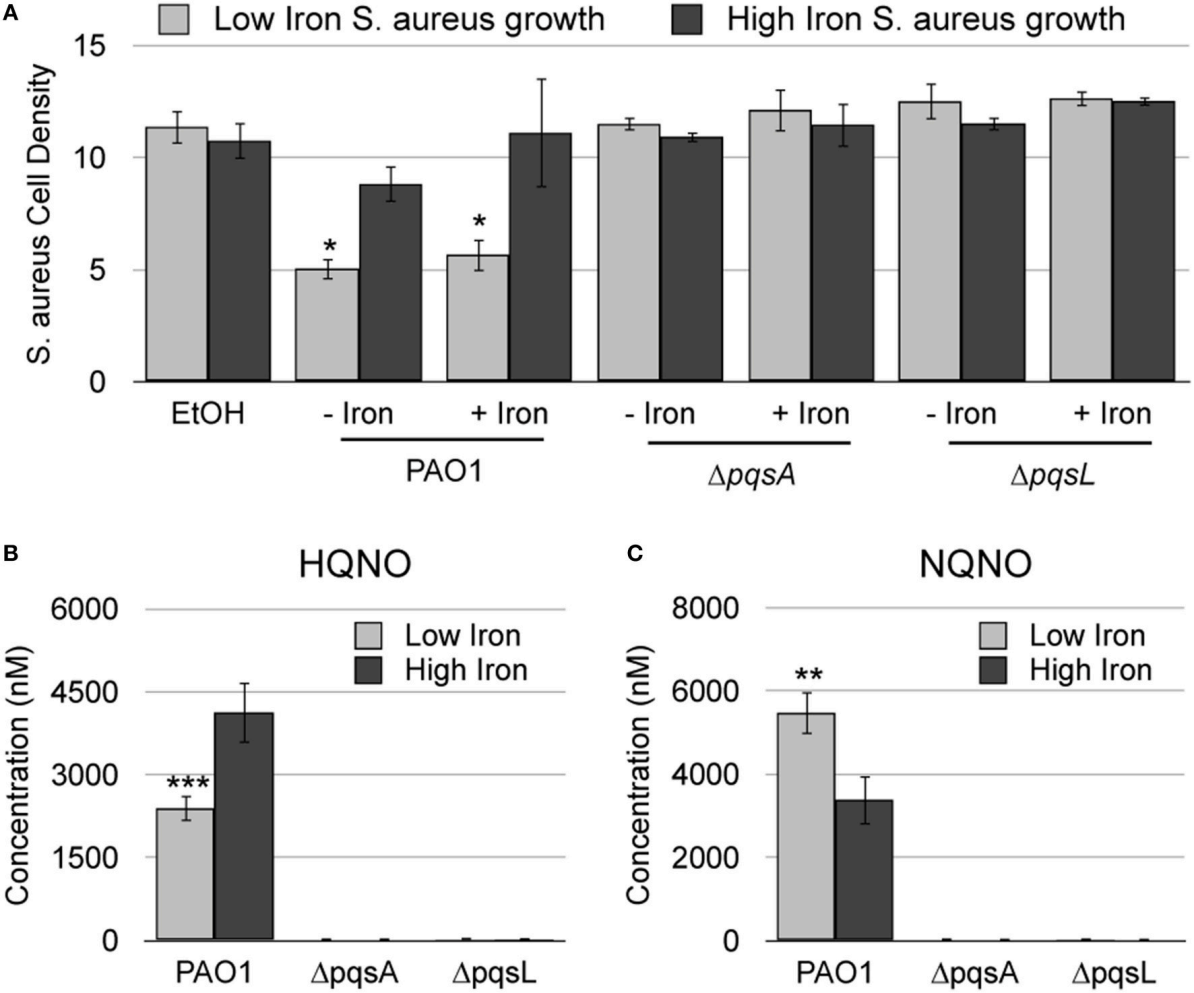
**AQNO-dependent antimicrobial activity against *S. aureus* is dependent upon iron depletion**. **(A)** *S. aureus* was grown overnight in DTSB supplemented with or without 100 μM FeCl_3_ and with the indicated *P. aeruginosa* AQ extracts, prepared as described in the Section Materials and Methods. **(B)** Culture supernatant extracts from the indicated *P. aeruginosa* strains were prepared and analyzed by LC-MS/MS as described in the Section Materials and Methods. Error bars indicate standard deviation of three **(A)** or five **(B)** biological replicates. Asterisks (*) indicate the following *p*-values as determined by two-tailed Student's *t*-test: ^*^p < 0.05, ^**^p < 0.005, ^***^p < 0.0005 when comparing low iron to high iron.

Previous analysis of a panel of *pqs* mutants demonstrated that antimicrobial activity of *P. aeruginosa* against *S. aureus* is dependent upon multiple AQs, including HQNO (Filkins et al., 2015; Nguyen et al., 2015). HQNO possesses innate antimicrobial activity against *S. aureus* due to its ability to bind to and inhibit the activity of cytochrome b (Lightbown and Jackson, 1956; Machan et al., 1992). Recent studies also demonstrate that both siderophores and AQNOs are required for antimicrobial activity against *S. aureus* during mixed biofilm growth (Filkins et al., 2015), indicating that iron may play a role in AQNO-mediated antimicrobial activity. We therefore sought to determine how iron supplementation affected the antimicrobial activity of AQNOs against *S. aureus*. Since *P. aeruginosa* produces several congeners of AQNOs, with varying alkyl chain lengths and levels of saturation, we chose to first analyze extracts of *P. aeruginosa* culture supernatants instead of assaying the effects of individual AQNO metabolites. AQs were extracted from wild type PAO1, the Δ*pqsA* mutant, which lacks production of all AQs, and the Δ*pqsL* mutant, which is specifically defective for AQNO production (Figure 1). *S. aureus* was then cultured in high or low iron media in the presence of each of these extracts. As expected, wild type PAO1 extracts substantially reduced *S. aureus* growth (by 50%) as compared to the solvent control (Figure 2A). This effect was eliminated when *S. aureus* cultures were subjected to either the Δ*pqsA* or Δ*pqsL* extracts (Figure 2A), demonstrating the specific role of AQNOs in inhibiting *S. aureus* growth in this assay. Notably, iron supplementation of *P. aeruginosa* cultures had no impact on the ability of the resulting extracts to inhibit *S. aureus* growth (Figure 2A). In contrast, iron depletion of *S. aureus* cultures significantly enhanced the antimicrobial activity of both the high and low iron PAO1 extracts (Figure 2A). This was not due to effects of the Pseudomonas growth medium, as extracted high or low iron DTSB media did not significantly affect *S. aureus* growth (Supplementary Figure S1). These data indicate that while iron does not affect on the production of AQNOs by *P. aeruginosa*, it does impact the susceptibility of *S. aureus* cultures to these metabolites.

**Figure 3:**
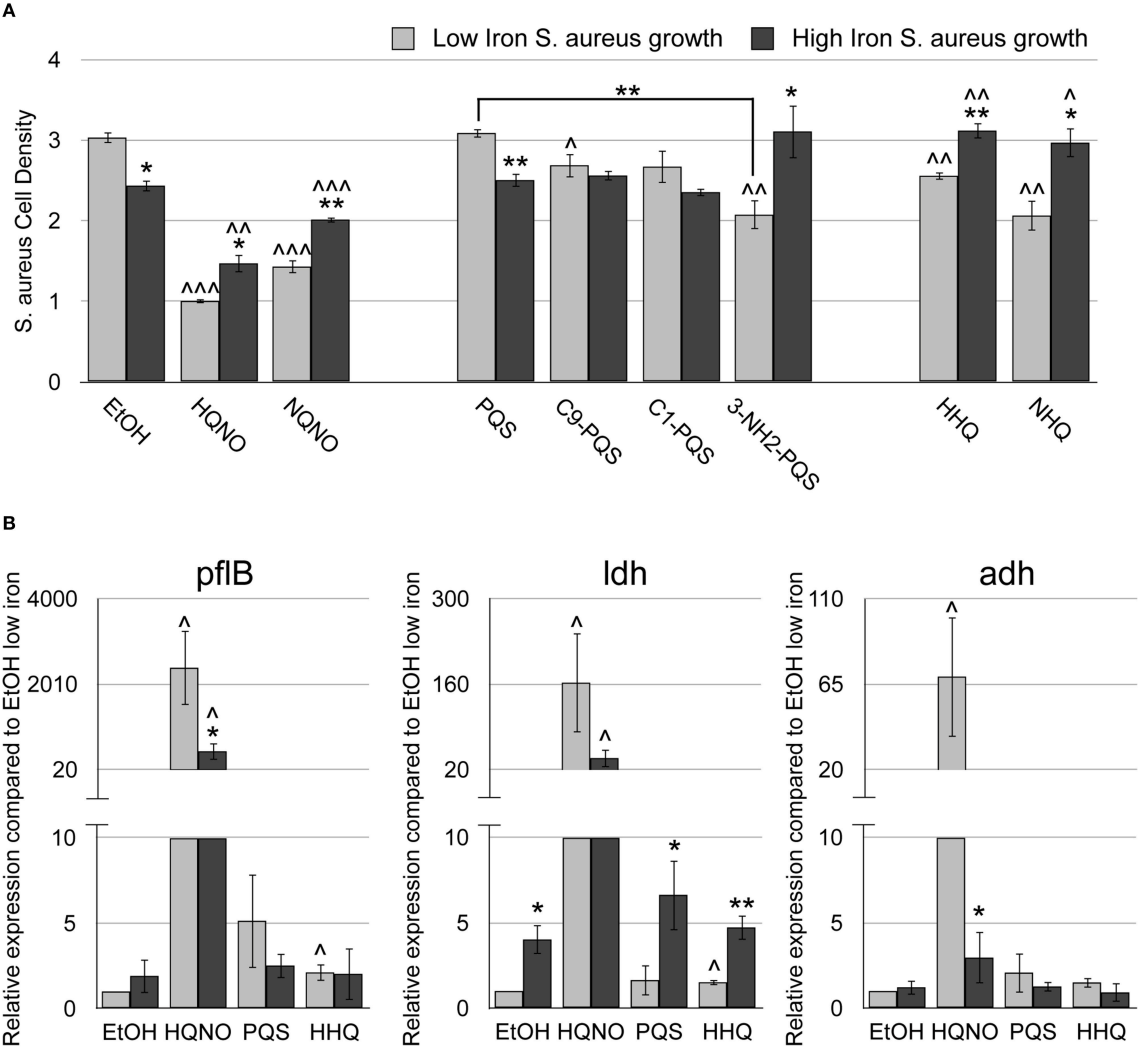
**HHQ possesses innate antimicrobial activity**. The methicillin-resistant *S. aureus* (MRSA) M2 strain was grown for 18 h at 37°C in DTSB, supplemented with or without 100 μM FeCl_3_ and 50 μM of the indicated AQs. **(A)** OD_600_ of overnight *S. aureus* cultures was measured. **(B)** *pflB, ldh*, and *adh* mRNA expression was measured by qRT-PCR of MRSA-M2 as described in Section Materials and Methods. Error bars indicate the standard deviation of three biological replicates. Asterisks (*) indicate the following *p*-values as determined by two-tailed Student's *t*-test when comparing low to high iron conditions: ^*^p < 0.05, ^**^p < 0.005. Carrots (∧) indicate the following *p*-values as determined by two-tailed Student's *t*-test when comparing AQ treatment to ethanol solvent (EtOH) alone: ^∧^*p* < 0.05, ^∧∧^*p* < 0.005, ^∧∧∧^*p* < 0.0005.

To directly determine if AQNO production is regulated by iron, we quantified AQNO levels in supernatants of *P. aeruginosa* cultures grown in low or high iron media using liquid chromatography tandem mass spectrometry (LC-MS/MS). For this analysis we selected HQNO, an AQNO congener with a C7 (heptyl) alkyl chain, and NQNO, an AQNO congener with a C9 (nonyl) alkyl chain. Strikingly, our results show that the impact of iron on AQNO production is dependent upon alkyl chain length: production of HQNO is repressed by iron depletion, while that of NQNO is induced by iron depletion (Figures 2B,C). These data further support the idea that iron depletion does not induce overall AQNO production by *P. aeruginosa*, and that antimicrobial activity of AQNOs is instead dependent upon iron depletion of *S. aureus* cultures. To directly test this idea, we subjected *S. aureus* low and high iron cultures to HQNO and NQNO synthesized as described previously (Ortori et al., 2011). While HQNO and NQNO suppressed growth of *S. aureus* when cultured in either high iron medium, iron depletion significantly enhanced the antimicrobial activity of these metabolites (Figure 3A). As expected, decreases in cell density correlated closely with changes in cellular respiration as determined by staining cultures with triphenyltetrazolium chloride (TTC), indicating that the effects of AQNOs are due to inhibition of cytochrome activity vs. loss of cell viability (Table S2). Combined, these results show that iron starvation sensitizes *S. aureus* cultures to the antimicrobial activity of AQNOs.

### HHQ displays innate antimicrobial activity against *S. aureus*

**Figure 4:**
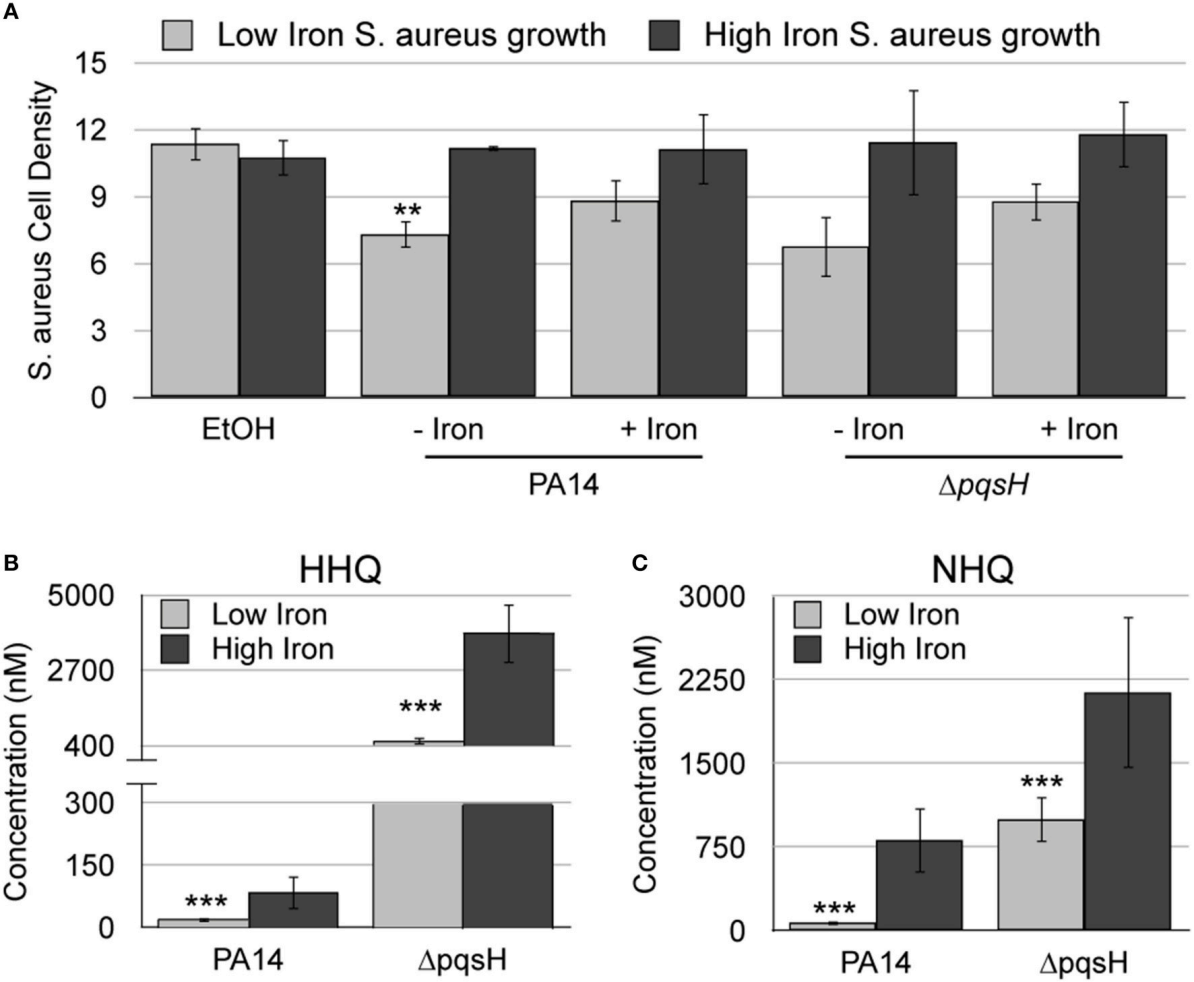
**HHQ is overproduced by the Δ*pqsH* mutant**. **(A)** *S. aureus* was grown overnight in DTSB supplemented with or without 100 μM FeCl_3_ and the indicated *P. aeruginosa* AQ extracts. OD_600_ of overnight *S. aureus* cultures was measured as described in Section Materials and Methods. **(B,C)** Culture supernatant extracts from the indicated *P. aeruginosa* strains, grown with or without 100 μM FeCl_3_ were analyzed by LC-MS/MS as described in the Section Materials and Methods. Error bars indicate standard deviation of three **(A)** and five **(B)** biological replicates. Asterisks (*) indicate the following *p*-values as determined by two-tailed Student's *t*-test: ^**^p < 0.005, ^***^p < 0.0005 when comparing low iron to high iron.

We next sought to determine how PQS and HHQ contribute to iron-regulated antimicrobial activity against *S. aureus*. PQS and HHQ both function as quorum sensing molecules, activating the expression of genes that contribute to *S. aureus* growth suppression (Deziel et al., 2004; Diggle et al., 2007). PQS is additionally able to chelate iron (Bredenbruch et al., 2006; Diggle et al., 2007), which could also contribute to growth suppression of *S. aureus*. Our previous work showed that *pqsH*, which is responsible for the conversion of HHQ to PQS (Figure 1), is not required for *S. aureus* growth suppression during co-culture (Nguyen et al., 2015). Deletion of *pqsH* also had no effect on the antimicrobial activity of culture supernatant extracts (Figure 4A). Combined, these results indicate the iron-chelation activity of PQS is dispensable for *S. aureus* growth suppression. Alternatively, it is possible that deletion of *pqsH* alters the production of AQ metabolites, resulting in enhanced antimicrobial activity by other mechanisms. We therefore used LC-MS/MS to quantify production of HHQ and HQNO, and related congeners, in the Δ*pqsH* mutant. While no significant changes in either HQNO or NQNO were observed (Supplementary Figures S2C,D), these results showed more than a 10-fold increase in HHQ and NHQ production in the Δ*pqsH* mutant as compared to the wild type strain in both high and low iron conditions (Figures 4B,C). It is therefore possible that increased production of HHQ suppresses defects in antimicrobial activity that are specific to loss of PQS.

We next determined if PQS, due to its ability to chelate iron, exhibits innate antistaphylococcal activity by subjecting *S. aureus* low and high iron cultures to PQS and related congeners (C9-PQS and C1-PQS). Interestingly, only C9-PQS caused a small but significant decrease in *S. aureus* culture density as compared the ethanol control (*p* < 0.05), and this effect was only observed in low iron (Figure 3A). To determine if this activity was due to iron chelation, we also treated *S. aureus* cultures with NHQ and HHQ, as well as a PQS mimic, 3-NH_2_-PQS. The replacement of an amino group for the hydroxyl group on the 3′ position of the quinolone allows for binding and activation of PqsR, but not iron chelation ability (Ilangovan et al., 2013). Surprisingly, 3-NH_2_-PQS, HHQ, and NHQ were all able to suppress growth of *S. aureus* in low iron (Figure 3A), indicating that the 3′ hydroxyl group of PQS inhibits the growth suppressive activity of these AQs. As observed for HQNO, the reduction in *S. aureus* culture density upon HHQ supplementation correlated with decrease in TTC staining, demonstrating a similar mechanism of growth inhibition (Table S2). Our analysis further shows that addition of either PQS or HHQ has an additive effect on *S. aureus* growth when provided in combination with HQNO (Supplementary Figure S3). The combination of all three AQs did not significantly alter the growth suppression of *S. aureus* compared to HQNO in combination with either PQS or HHQ (Supplementary Figure S3). These data demonstrate a novel role for these signaling molecules in mediating antimicrobial activity against *S. aureus*.

### HQNO and HHQ induce fermentation pathways in *S. aureus*

A recent report by Filkins et al. demonstrated that co-culture with *P. aeruginosa* significantly altered *S. aureus* gene expression in an AQNO-dependent manner (Filkins et al., 2015). The most up-regulated genes were associated with fermentation pathways of *S. aureus* including formate acetyltransferase (*pflB*), L-lactate dehydrogenase (*ldh*), and alcohol dehydrogenase (*adh*). To determine whether synthesized AQNOs were able to induce these same genes, we performed real time PCR on *S. aureus* cultures grown with HQNO in DTSB supplemented with or without iron. HQNO induced *pflB, ldh*, and *adh* gene expression 100–1000 fold in low iron compared to the ethanol control, similar to that observed by Filkins et al. (Figure 3B). Strikingly, HQNO's ability to induce *pflB, ldh*, and *adh* expression in *S. aureus* was reduced in iron-replete conditions (Figure 3B). Thus, iron levels significantly impact on the ability of HQNO to induce fermentative metabolism genes in *S. aureus*.

The studies by Filkins et al. were conducted with co-cultures of *S. aureus* with *P. aeruginosa*, or in the presence of *P. aeruginosa* culture extracts (Filkins et al., 2015), leaving the possibility that other *P. aeruginosa* metabolites contribute to the AQNO-dependent shift in *S. aureus* metabolism. Since we showed that HHQ possesses innate antimicrobial activity against *S. aureus*, we assayed gene expression of *S. aureus* treated with HHQ or PQS to determine if either could drive *S. aureus* to fermentative metabolism. While the effects of HHQ were not as robust as that of HQNO, HHQ did cause a small but significant induction of *pflB* and *ldh* expression as compared to the ethanol control (Figure 3B). Moreover, induction of these genes by HHQ was only noted under iron-depleted conditions (Figure 3B—light gray bars). In contrast, PQS had no significant effect on *pflB, ldh*, or *adh* expression in either high or low iron condition compared to the ethanol control (Figure 3B). Notably, the ability of the individual AQ metabolites to induce fermentative gene expression correlated with their growth suppressive activity against *S. aureus* (Figure 3A). Thus, while HQNO appears to be the major metabolite responsible for increasing expression of *S. aureus* fermentative genes and growth suppressive activity, HHQ is also capable of altering *S. aureus* gene expression related to fermentative metabolism and inhibiting growth of *S. aureus*. Moreover, the sensitivity of *S. aureus* cultures to each of these metabolites is enhanced by iron depletion, presenting a novel means by which iron affects antimicrobial activity of *P. aeruginosa* against *S. aureus*.

### Iron-regulated antimicrobial activity is exhibited by multiple CF isolates

**Figure 5:**
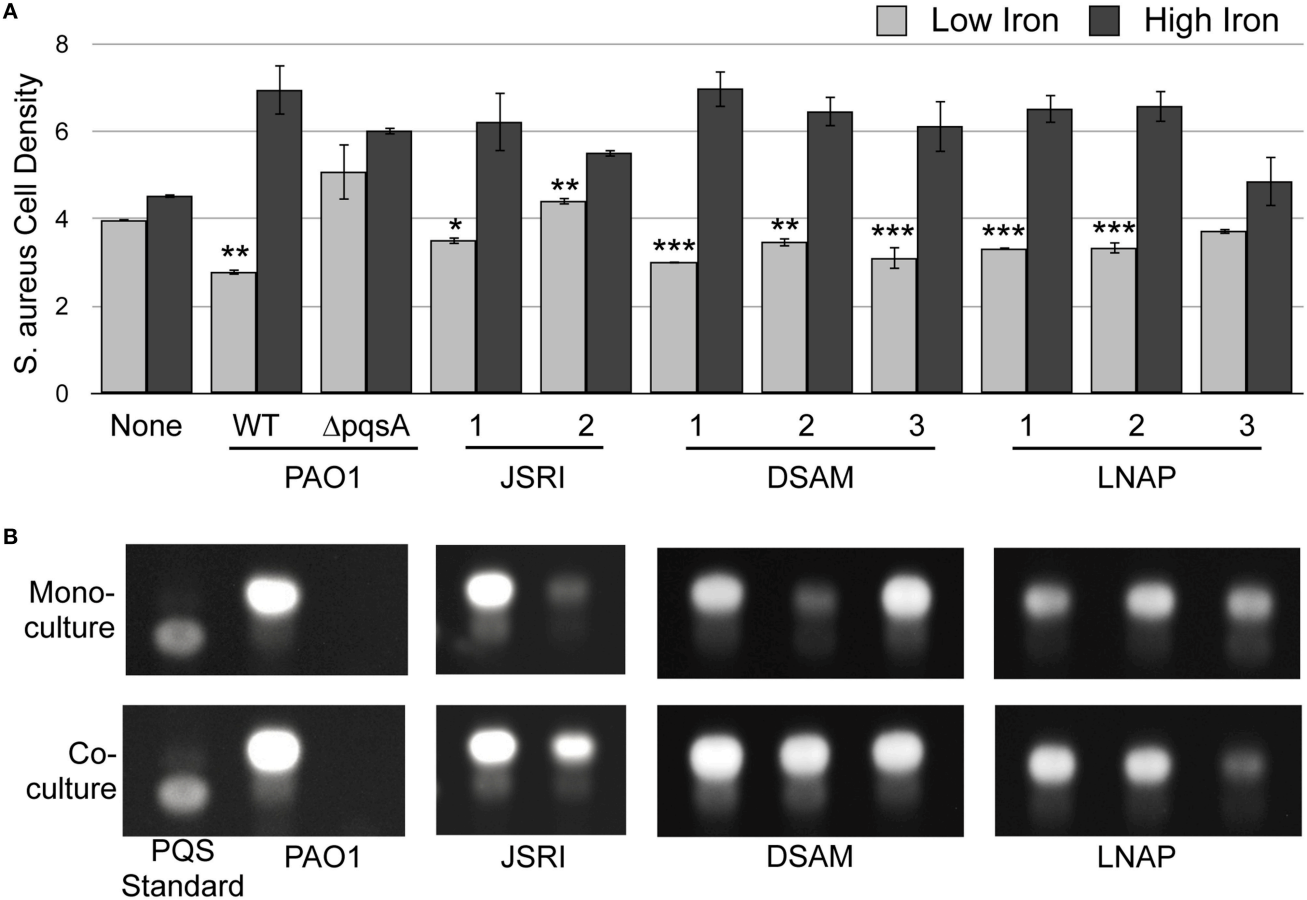
***S. aureus* restores PQS production to several CF isolates of *P. aeruginosa*. (A)** *S. aureus* cell density was measured spectroscopically as OD_630_ after co-culture with the indicated *P. aeruginosa* strains in transwell cell culture plates as described in Section Materials and Methods. Error bars indicate standard deviation of three biological replicates. (*) Asterisks indicate the following *p*-values as determined by a two-tailed Student's *t*-test: ^*^p < 0.05, ^**^p < 0.005, ^***^p < 0.005 when comparing low iron to high iron. **(B)** TLC of *P. aeruginosa* laboratory strains and clinical CF isolates. *P. aeruginosa* strains were grown with or without *S. aureus* as indicated, and culture supernatant extracts were prepared and analyzed by as described in Section Materials and Methods. Images are representative of three biological replicates.

Our previous studies showed that two clonal, longitudinally-isolated strains of *P. aeruginosa* from an individual CF patient exhibited iron-regulated antimicrobial activity against *S. aureus* (Nguyen et al., 2015). To determine if other CF isolates are capable of this activity, we examined clonal, longitudinally-isolated CF strains of *P. aeruginosa* from two additional patients: DSAM (DSAM-1, DSAM-2, and DSAM-3) and LNAP (LNAP-1, LNAP-2, and LNAP-3). These strains were isolated from CF patients ranging from age 2 to 22, providing a broad view of *P. aeruginosa* isolates from multiple stages of CF lung infection (Table 1). *S. aureus* was grown in co-culture with each CF isolate of *P. aeruginosa*, separated by a transwell membrane as previously described (Nguyen et al., 2015), in either high or low iron. With the exception of LNAP-3, each of these isolates exhibited iron-regulated antimicrobial activity against *S. aureus* (Figure 5A). As previously shown, the clonal JSRI-1 and JSRI-2 CF isolates also both exhibited iron-regulated antimicrobial activity against *S. aureus* (Figure 5A). These data indicate that iron-regulated antimicrobial activity against *S. aureus* is largely conserved by *P. aeruginosa* throughout CF lung infection, although variations in this activity between individual isolates does exist.

### PQS-deficient CF isolates increase production of HHQ

We next determined the potential role of individual AQs produced by CF isolates in iron-regulated antimicrobial activity against *S. aureus*. We previously found that PQS production of the JSRI-2 isolate is reduced as compared to the JSRI-1 isolate (Nguyen et al., 2014). However, co-culture with *S. aureus* restores PQS production to JSRI-2, potentially allowing for antimicrobial activity observed by this strain (Nguyen et al., 2015). To determine if PQS production similarly correlates with antimicrobial activity in the LNAP and DSAM CF isolates (Figure 5A), we performed thin layer chromatography (TLC) on culture supernatants from each isolate, grown either in mono-culture or co-culture with *S. aureus*. Similar to JSRI-2, DSAM-2 showed weak PQS production in monoculture (Figure 5B), yet was still able to exert antimicrobial activity against *S. aureus* (Figure 5A). Also similar to JSRI-2, we found that PQS production of DSAM-2 was restored to this isolate when co-cultured with *S. aureus* (Figure 5B). We also noted weak PQS production by LNAP-3 in mono-culture, which was not restored by co-culture with *S. aureus* (Figure 5B). Thus, co-culture with *S. aureus* restores PQS production to certain *P. aeruginosa* CF isolates, potentially allowing for iron-regulated antimicrobial activity against *S. aureus*.

Since the data above and our previous work shows that multiple AQs contribute to antimicrobial activity by *P. aeruginosa* laboratory strains (Nguyen et al., 2014), we next examined production of HQNO and HHQ by the JSRI, DSAM, and LNAP isolates. Similar to what was observed for PQS, production of both HQNO and NQNO was reduced in the JSRI-2, DSAM-2, and LNAP-3 isolates as compared to clonal isolates from the same patient (Table 2). HHQ and NHQ production were also reduced in JSRI-2 as compared to JSRI-1 (Table 2). In contrast, DSAM-2 and LNAP-3 showed increased production of HHQ and NHQ as compared to clonal isolates from the same patient (Table 2), similar to that observed in the PA14 Δ*pqsH* mutant (Figures 4B,C). Combined, these results suggest that JSRI-2 has an overall defect in AQ metabolism, while DSAM-2 and LNAP-3 are specifically defective for PQS and AQNO production.

**Table 2:** **Quantification of AQ metabolites in CF isolates**.

**Strain**	**Concentration (nM)**^a^ **of:**			
	**HQNO**	**NQNO**	**HHQ**	**NHQ**
JSRI-1	2629.7 ± 580.7	5330.0 ± 1150.1	80.7 ± 9.6	285.1 ± 79.5
JSRI-2	22.8 ± 7.0^***^	3162.4 ± 1299.6^*^	17.5 ± 8.9^***^	26.7 ± 12.9^***^
DSAM-1	4539.4 ± 720.6	8342.4 ± 1657.1	22.7 ± 2.8	172.2 ± 27.9
DSAM-2	3073.4 ± 429.2^*^	4948.5 ± 736.2^*^	903.0 ± 455.4^***^	515.9 ± 63.7^***^
DSAM-3	4278.3 ± 528.2	7957.3 ± 944.4	25.6 ± 10.6	275.8 ± 114.3
LNAP-1	2770.2 ± 863.4	5699.6 ± 1718.7	15.6 ± 6.1	42.5 ± 16.9
LNAP-2	2996.7 ± 876.2	5969.8 ± 1642.0	17.8 ± 5.5	46.3 ± 14.0
LNAP-2	1101.0 ± 280.2^*^	2098.3 ± 624.6^**^	192.4 ± 83.3^**^	674.2 ± 132.8^***^

a Determined by LC-MS/MS as described in the Section Materials and Methods. Standard deviation is from five biological replicates. Asterisks (^*^) indicate the following p-values as determined by two-tailed Student's t-test when comparing to the parent strain in each CF series:

* *p < 0.05*,

** *p < 0.005*,

*** *p < 0.0005*.

To determine the molecular basis for altered AQ metabolite production by the JSRI-2, DSAM-2, and LNAP-3 isolates, we performed real time PCR of the *pqsA* and *pqsH* genes. These analyses show that *pqsA* gene expression is significantly reduced in the JSRI-2 isolate as compared to JSRI-1, while expression of *pqsH* is increased in the JSRI-2 isolate (Table 3), supporting the hypothesis that JSRI-2 exhibits an overall defect in AQ metabolism. In contrast, *pqsA* expression is maintained in DSAM-2 and increased in LNAP-3 as compared to their clonal isolates (Table 3), while *pqsH* expression is substantially reduced in these strains (Table 3). Thus, defects in the terminal step of PQS synthesis in these isolates correlates with an accumulation of HHQ intermediates. Surprisingly, DSAM-3, which showed no defects in PQS, HHQ, or HQNO production, exhibited significantly reduced expression of both *pqsA* and *pqsH* (Table 3). The rationale for this apparent paradox is not clear. Overall, these studies suggest that the multifactorial nature of AQ-dependent antimicrobial activity allows *P. aeruginosa* to maintain this activity amid changes in AQ metabolites during CF lung infection.

**Table 3:** ***pqs* gene expression by PQS-deficient CF isolates**.

**Strain**	**Relative expression**^a^ **of:**	
	**pqsA**	**pqsH**
JSRI-1	1.00	1.00
JSRI-2	0.25 ± 0.03^***^	3.06 ± 0.39^***^
DSAM-1	1.00	1.00
DSAM-2	1.01 ± 0.24	0.04 ± 0.02^***^
DSAM-3	0.13 ± 0.10^***^	0.10 ± 0.32^***^
LNAP-1	1.00	1.00
LNAP-2	1.04 ± 0.37	0.94 ± 0.24
LNAP-2	5.26 ± 2.42	0.08 ± 0.02^***^

a Determined by qRT-PCR as described in the Section Materials and Methods. Standard deviation is of three biological replicates. Asterisks (^*^) indicate the following p-values as determined by two-tailed Student's t-test when comparing to the parent strain in each CF series:

*** *p < 0.0005*.

## Discussion

The decline of *S. aureus* and eventual dominance of *P. aeruginosa* is a common occurrence in the lungs of CF patients (Cystic Fibrosis Foundation, 2014). However, the mechanisms underlying this shift are still not well understood. AQs likely contribute to the ability of *P. aeruginosa* to outcompete *S. aureus* in this environment, as several AQs exhibit growth suppressive properties (Hoffman et al., 2006; Heeb et al., 2011). Furthermore, AQs have been found in the sputum, plasma, and urine of CF patients, highlighting their potential importance in CF infection (Barr et al., 2015). In this study, we show that in addition to its roles as a quorum sensing molecule, HHQ possesses innate antimicrobial activity against *S. aureus*. Additionally, we show that *S. aureus* is sensitized to the antimicrobial activity of both HHQ and HQNO when grown under iron limiting environments. Using a combination of LC-MS/MS and gene expression analysis, we provide evidence for how CF isolates of *P. aeruginosa* maintain antimicrobial activity during chronic lung infection, despite substantial changes in AQ metabolism. Combined, these results yield many novel insights into how iron affects the progression of *P. aeruginosa-S. aureus* co-infections, demonstrating the complexity of this dynamic microbial interaction.

We previously showed that iron-regulated antimicrobial activity is dependent in part upon AQNOs (Nguyen et al., 2015). Here, we show that iron depletion enhances AQNO-dependent antimicrobial activity against *S. aureus*, presenting a novel mechanism by which iron can affect AQ-dependent antimicrobial activity. These are particularly interesting results in light of a recent study from Filkins et al. showing that antimicrobial activity of *P. aeruginosa* against *S. aureus* in mixed biofilms is dependent upon siderophore production, which can similarly reduce extracellular iron levels (Filkins et al., 2015). What remains unknown is the precise mechanism of how AQNOs and iron depletion induce *S. aureus* to shift toward fermentative metabolism. Respiratory metabolism is heavily dependent upon heme- and iron-cofactored enzymes, and iron starvation has previously been shown to redirect *S. aureus* from respiratory to fermentative metabolism in a manner dependent upon the ferric uptake regulator (Fur; Friedman et al., 2006). Thus, iron depletion may exert an additive effect on *S. aureus* respiratory capabilities in the presence of AQNOs. The implications of this phenomenon for CF disease are particularly interesting, as inhibition of *S. aureus* respiration by HQNO selects for small colony variants (SCVs), which rely on fermentative metabolism and display increased tolerance to multiple antimicrobials (Pan et al., 2002; Proctor et al., 2006, 2014; Lechner et al., 2012; Wood et al., 2013). Notably, the presence of HQNO under our culture conditions reversed the response of *S. aureus* to environmental iron levels (Figure 3A), indicating iron supplementation can help *S. aureus* compensate for the deleterious effects of HQNO on respiratory metabolism. Identifying the specific factors of *S. aureus* that are responsible for regulating this response to HQNO and iron starvation will be critical for understanding the full scope of this microbial interaction.

While iron depletion enhances production of the C9 congener of PQS (Nguyen et al., 2014), the precise contribution of this and related AQ metabolites to overall antimicrobial activity remains elusive. In addition to its role as a quorum signaling molecule, PQS also exhibits iron chelating activity (Bredenbruch et al., 2006; Diggle et al., 2007), which could contribute to antimicrobial activity against *S. aureus*. However, our previous work suggested that PQS is dispensable for this activity, as a Δ*pqsH* mutant exhibited antimicrobial activity similar to its wild type parent (Nguyen et al., 2015). While HHQ is not as active as PQS as a quorum signaling molecule (Xiao et al., 2006; Diggle et al., 2007), we noted a substantial increase in HHQ and NHQ production in the Δ*pqsH* mutant (Figures 4B,C). The current work further shows that HHQ possesses innate growth suppressive activity against *S. aureus* and can induce expression of *S. aureus* fermentative genes when environmental iron is limiting (Figure 3). While not nearly as active of an antimicrobial as HQNO, these data suggest that HHQ can inhibit *S. aureus* growth by means similar to HQNO, and that overproduction of this metabolite may be able to compensate for *P. aeruginosa* defects in production of PQS and other AQ metabolites.

*P. aeruginosa* undergoes substantial changes as it adapts to the CF lung environment, including the loss of several virulence-related genes (Smith et al., 2006; Dettman et al., 2013; Huse et al., 2013; Marvig et al., 2014). Our previous studies showed that PQS production was reduced in at least one CF isolate (JSRI-2) over time in the CF lung (Nguyen et al., 2014). Despite this reduction, the JSRI-2 isolate retained the ability to mediate iron-regulated antimicrobial activity, potentially due to the finding that co-culture with *S. aureus* restored PQS production to this isolate (Nguyen et al., 2015). In the current study, analysis of clonal and longitudinal isolates from additional CF patients has provided further models into how *P. aeruginosa* retains antimicrobial activity during CF lung infection. Similar to the isogenic Δ*pqsH* mutant, overproduction of HHQ by CF isolates has the potential to compensate for loss of PQS production in these isolates, and thus allow *P. aeruginosa* to retain antimicrobial activity against *S. aureus*.

It is important to note that the isolates analyzed in this study represent only a small subset of the *P. aeruginosa* community in the CF lung, which has been shown to be highly heterogeneous (Dettman et al., 2013; Winstanley et al., 2016). Thus, it is possible that AQ metabolites produced by other members of the community allowed for the emergence of metabolic cheats, as has been noted in multiple studies (Andersen et al., 2015; Ross-Gillespie et al., 2015). The finding that nearly all of the CF isolates we have thus far tested exhibit iron-regulated antimicrobial activity against *S. aureus* points to this phenomenon being independent of the cheater paradigm, at least in the context of mixed *P. aeruginosa* communities. Instead, it is likely that the multifactorial nature of iron-regulated antimicrobial activity allows it to persist in spite of changes incurred to AQ metabolites. It is also important to note that, with the exception of the transwell co-culture assays, the experiments in this study were performed on shaking, planktonic cultures, while microbial growth in the CF lung occurs in biofilms. It is possible that the antimicrobial activity of individual AQs against *S. aureus* biofilms could differ from that of planktonic cells to some extent. However, previous work by Filkins et al. has shown that both AQ production and iron availability contribute to *P. aeruginosa'*s antimicrobial activity against *S. aureus* in biofilms. Thus, the results that we show here are likely translatable to mixed biofilm growth of these two species.

Iron is an essential nutrient for both *P. aeruginosa* and *S. aureus* and is increasingly appreciated as a critical mediator of CF lung disease (Cassat and Skaar, 2013; Barnabie and Whiteley, 2015; Bouvier, 2016). The finding that iron modulates antimicrobial activity of multiple *P. aeruginosa* CF isolates further indicates the importance of this essential nutrient in progression of CF lung disease. While siderophores are critical for antimicrobial activity in laboratory strains of *P. aeruginosa* (Filkins et al., 2015), siderophore-mediated iron uptake is less likely to be important for this process in the CF lung due to reduced dependence on these systems as disease progresses (Marvig et al., 2014; Nguyen et al., 2014). Determining how primary clinical isolates of *P. aeruginosa* mediate antimicrobial activity in the context of CF lung infection will therefore require further studies of iron uptake and regulation by primary CF isolates, and well as determining the impact of relevant host immune factors that sequester host iron sources. Most importantly, this work demonstrates the importance of considering iron and other essential nutrients when studying microbial interactions in the context of human disease.

## Author contributions

AN, JJ, MC, PW, MK, AO contributed substantially to the conception/design, acquisition, and analysis of this work. AN, JJ, MC, PW, MK, AO revised the work for intellectual content and approved the final version for publication. AN, JJ, MC, PW, MK, AO agree to be accountable for all aspects of this work.

### Conflict of interest statement

The authors declare that the research was conducted in the absence of any commercial or financial relationships that could be construed as a potential conflict of interest.
