# Supplementary material for: Cystic Fibrosis Isolates of Pseudomonas aeruginosa Retain Iron-Regulated Antimicrobial Activity against Staphylococcus aureus through the Action of Multiple Alkylquinolones
Source: Front Microbiol. 2016 Jul 27;7:1171. doi: 10.3389/fmicb.2016.01171 (PMC4961689; doi:10.3389/fmicb.2016.01171)

Table S1. Real time PCR primers and probes used in this study.

| **Oligonucleotide** | **Sequence 5’ – 3’** |
| --- | --- |
| *Primers* |  |
| *oprF.*for | GCG TTC GCA ACA TGA AGA AC |
| *oprF.*rev | CTT CTT GTT GCC GGT TTC GTA |
| *pqsA*.for | CCT CGA TTT CGA TCC CGA TAC |
| *pqsA*.rev | TGG CCT GGG AGA GAA TGT AGG |
| *pqsH*.for | TCG AGT TCA TCA GGA AGC AAT C |
| *pqsH*.rev | CGA GGG TAT TCC TCA GCC AGA |
| *pqsL*.for | CGA AGC GAC CGT CGA GAT |
| *pqsL*.rev | TAG GAG GCG ATA CCG TCG G |
| *rpoB*.for | TTC AGC AGC GAC AGC ATG TAT |
| *rpoB*.rev | CTG CAA CGA TTG GAC GTT GG |
| *pflB*.for | AGA CGG ACG TAA AGC TGG CG |
| *pflB*.rev | TGC ACC TTT TTG GTCC ACG GC |
| *ldh.*for | TGC TGG TGC TGC ACA AAA AAC |
| *ldh.*rev | CGT GGC GCA ACA TCG AAC G |
| *adh.*for | AAG AAC GGA ACA GCC TCA AAT T |
| *adh.*rev | AAC CAC CAC CAA GTG CAA TGA |
| *Probes* |  |
| *oprF* | CGG TGA GTA CCA TGA CGT TCG TGG C |
| *pqsA* | CAC TAT CGG GGC CAG ACT CTC AGC C |
| *pqsH* | CTT GGT CAG TGG GAA TCG CCC TCC |
| *pqsL* | CGA GCG CCA CGC GAT CGA C |
| *rpoB* | ACG GCG TTG AGC ATG AAG GTG AAT TAG ATC |
| *pflB* | CCA TTT GCA CCA GGT GCA AAC CC |
| *ldh* | CAA ATC AAT TGT TGG TGA AGT AAT GGC ATC AA |
| *adh* | TAG CGA AGT CGA ACC GAA CCC ATC A |

**Table S2. Culture density of AQ-treated *S. aureus* correlates with metabolic activity*.***

| **AQ** | **Low Iron** |  | **High Iron** |  |
| --- | --- | --- | --- | --- |
|  | **OD_600_** | **OD_510_** | **OD_600_** | **OD_510_** |
| EtOH | 3.25 ± 0.58 | 3.05 ± 0.30 | 2.99 ± 0.44 | 2.82 ± 0.47 |
| HQNO | 1.05 ± 0.14* | 0.78 ± 0.11** | 1.59 ± 0.11* | 1.57 ± 0.21* |
| PQS | 2.54 ± 0.14 | 2.40 ± 0.30 | 2.55 ± 0.25 | 2.40 ± 0.38 |
| HHQ | 2.29 ± 0.02* | 1.95 ± 0.35* | 3.37 ± 0.27 | 3.33 ± 0.68 |
| Determined by TTC assay. The methicillin-resistant *S. aureus* (MRSA) M2 strain was grown for 18 hours at 37˚C in DTSB, supplemented with or without 100 µM FeCl_3_ and 50 µM of the indicated AQs. OD_600_ was measured. 0.1% 2,3,5-triphenyltetrazolium chloride (TTC) was added to cultures, cells were spun down, resuspended in 50% ethanol and OD_510_ was measured. Standard deviations are of three biological replicates. Asterisks (*) indicate the following *p* values as determined by two-tailed Student’s *t* test when comparing to EtOH control: * *p* < 0.05, ** *p* < 0.005. | | | | |

**FIGURES**

**Supplementary Figure 1. Extracted DTSB media and EtOH controls behave similarly.** The methicillin-resistant *S. aureus* (MRSA) M2 strain was grown for 18 hours at 37˚C in DTSB, supplemented with or without 100 µM FeCl_3_ and 1 µL of 100% ethyl alcohol or extracted DTSB media resuspended in 100% ethyl alcohol as indicated. OD_600_ of overnight *S. aureus* cultures was measured as described in materials and methods. Error bars indicate the standard deviation of three biological replicates. Results indicate no significance (*p* > 0.05) in any conditions as determined by two-tailed Student’s *t* test.

**
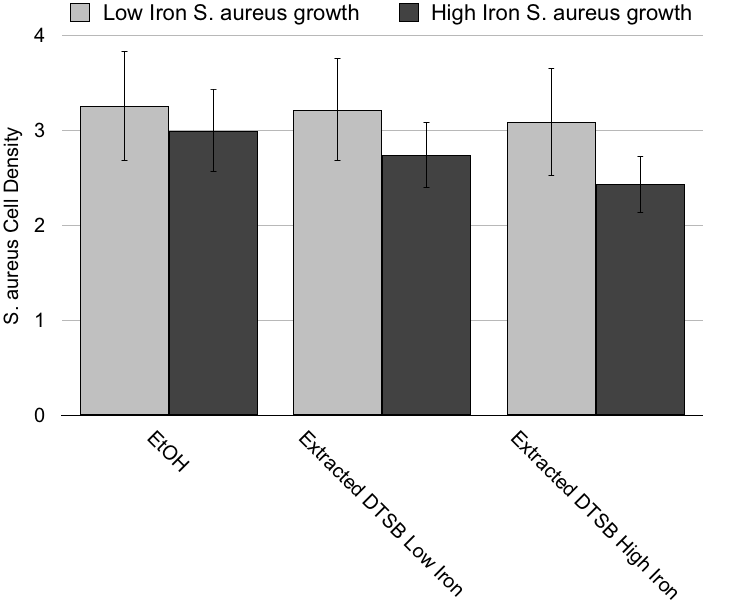
**

**Supplementary Figure 2. LC-MS/MS of AQ metabolites in PQS biosynthetic mutants.** The indicated *P. aeruginosa* strains were grown in DTSB and culture supernatant were extracted as described in the materials and methods. Extracts were analyzed by LC-MS/MS for (A) HHQ, (B) NHQ, (C) HQNO, and (D) NQNO as described. Error bars indicate standard deviation of five biological replicates. Asterisks (*) indicate the following *p* values as determined by two-tailed Student’s *t* test: * *p* < 0.05, *** *p* < 0.0005 when comparing back to the parent strain (PAO1 or PA14).


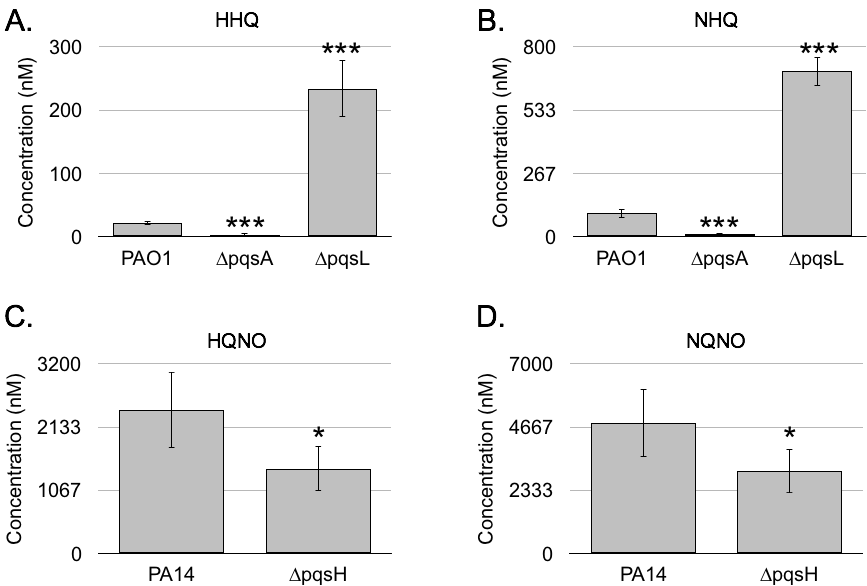


**Supplementary Figure 3. PQS/HHQ and HQNO growth suppressive effects are additive*.*** The methicillin-resistant *S. aureus* (MRSA) M2 strain was grown for 18 hours at 37˚C in DTSB, supplemented with or without 100 µM FeCl_3_ and 50 µM of the indicated AQs. OD_600_ of overnight *S. aureus* cultures was measured as described in materials and methods. Error bars indicate the standard deviation of three biological replicates. Asterisks (*) indicate the following *p* values as determined by two-tailed Student’s *t* test when comparing low or high iron conditions: * *p* < 0.05, ** *p* < 0.005, *** *p* < 0.0005. Carrots (^) indicate the following *p* values as determined by two-tailed Student’s *t* test when comparing AQ treatment to ethanol solvent (EtOH) alone: ^ *p* < 0.05, ^^^ *p* < 0.0005.


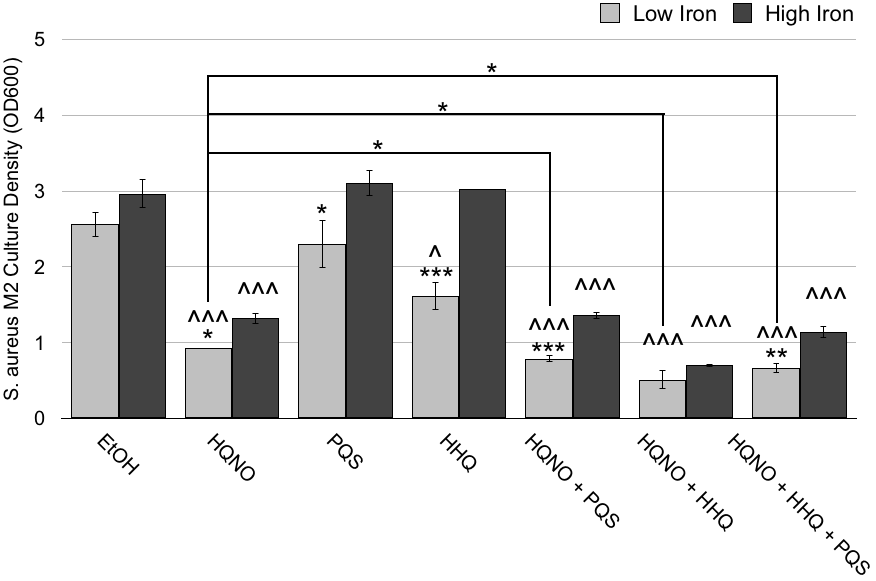

Supplement: Supplementary file 1 [file DataSheet1.DOC]
